# Culture Adaptation Alters Transcriptional Hierarchies among Single Human Embryonic Stem Cells Reflecting Altered Patterns of Differentiation

**DOI:** 10.1371/journal.pone.0123467

**Published:** 2015-04-14

**Authors:** Paul J. Gokhale, Janice K. Au-Young, SriVidya Dadi, David N. Keys, Neil J. Harrison, Mark Jones, Shamit Soneji, Tariq Enver, Jon K. Sherlock, Peter W. Andrews

**Affiliations:** 1 Centre for Stem Cell Biology, Department of Biomedical Science, University of Sheffield, Sheffield, United Kingdom; 2 ThermoFisher, Foster City, California, United States of America; 3 Weatherall Institute of Molecular Medicine, University of Oxford, Oxford, United Kingdom; University of Southern California, UNITED STATES

## Abstract

We have used single cell transcriptome analysis to re-examine the substates of early passage, karyotypically Normal, and late passage, karyotypically Abnormal (‘Culture Adapted’) human embryonic stem cells characterized by differential expression of the cell surface marker antigen, SSEA3. The results confirmed that culture adaptation is associated with alterations to the dynamics of the SSEA3(+) and SSEA3(-) substates of these cells, with SSEA3(-) Adapted cells remaining within the stem cell compartment whereas the SSEA3(-) Normal cells appear to have differentiated. However, the single cell data reveal that these substates are characterized by further heterogeneity that changes on culture adaptation. Notably the Adapted population includes cells with a transcriptome substate suggestive of a shift to a more naïve-like phenotype in contrast to the cells of the Normal population. Further, a subset of the Normal SSEA3(+) cells expresses genes typical of endoderm differentiation, despite also expressing the undifferentiated stem cell genes, *POU5F1 (OCT4)* and *NANOG*, whereas such apparently lineage-primed cells are absent from the Adapted population. These results suggest that the selective growth advantage gained by genetically variant, culture adapted human embryonic stem cells may derive in part from a changed substate structure that influences their propensity for differentiation.

## Introduction

Populations of stem cells are subject to selection pressure for variants that favor self-renewal over the alternative fates of differentiation or cell death [1] [2]. Indeed, human embryonic stem (ES) cells often undergo culture adaptation after prolonged periods, showing altered growth characteristics, such as increased population doubling times and plating efficiencies [3, 4]. At the same time, they may acquire genetic or epigenetic changes [4–7]. The most commonly observed changes are manifest cytogenetically as non-random gains of chromosome fragments, notably involving chromosome 1, 12, 17 and 20, changes that also characterize embryonal carcinoma (EC) cells, the malignant counterparts of ES cells and the stem cells of teratocarcinomas [1, 7]. The non-random nature of the karyotypic changes, their association with pluripotent stem cells, and the rapid overgrowth of genetically variant cells over several passages [2], all argue for selection based on growth advantages derived from alterations to the genome. The commonality of the cytogenetic changes seen in human ES cells in culture and EC cells from teratocarcinomas suggests that culture adaptation and aneuploidy of ES cells *in vitro* could provide a paradigm for some aspects of tumor progression in cancers that involve a stem cell population.

The growth advantage of the culture adapted cells, which is apparent from their enhanced clonogenic capacity, could arise from a reduced tendency to undergo apoptosis [8], or to differentiate, or from an altered pattern of differentiation [9]. In a previous study comparing early passage, ‘normal’, and late passage, culture adapted human ES cells, we found that human ES cells within the stem cell compartment can exist in alternative substates defined by differential expression of the ES cell surface marker, SSEA3 [4]: in the early passage cultures, clonogenic cells were present primarily in the SSEA3(+) subset, whereas clonogenic cells were found in both the SSEA3(+) and SSEA3(-) subsets isolated from a culture adapted subline. These results, supported by microarray transcriptome data, suggested that during differentiation human ES cells first lose expression of SSEA3 and subsequently commit to differentiate, while culture adaptation raises a barrier that decreases the probability that cells go on and differentiate after losing SSEA3 expression. As a result, culture adaptation appears to ‘trap’ stem cells in the SSEA3(-) substate, allowing them to revert to an SSEA3(+) substate. Other studies have similarly suggested that pluripotent stem cells can exist in interconvertible substates, defined by other surface antigens [10] or expression of transcription factors like NANOG [11], STELLA [12], REX1 [13] and HEX [14].

The existence of substates within the stem cell compartment raises the question of whether a stem cell in a particular substate might be biased towards certain lineages before commitment to differentiation [15]. Such biases have been suggested in a promyelocytic leukemic stem cell [16], in neural differentiation of a human EC cell line NTERA2 [17], and during hematopoietic differentiation of human ES cells [18]. The molecular basis for such biases remains largely unknown, although recently WNT signaling has been implicated in biasing human ES cell self-renewal and lineage potential [19]. Nevertheless, the phenomenon of multi-lineage priming whereby transcripts of genes pertinent to specific pathways of differentiation can be detected in individual, undifferentiated cells, has been long known in hematopoietic stem cells [20] [21] while, in naïve pluripotent murine stem cells, there is evidence of transcriptional pausing, indicative of many loci being primed for transcription [22]. Thus, one possibility is that the growth advantages of culture adapted ES cells can arise from alterations to the dynamics of the various lineage primed substates in which they may exist, in turn affecting their patterns of differentiation. To address this we have now re-examined the SSEA3(+) and (-) subsets that we previously identified in the early passage, normal, and late passage, culture adapted, sublines of H7 human ES cells [4], to determine whether at a single cell level there is evidence of lineage priming, and whether this is influenced by culture adaptation in a way that might contribute to a growth advantage for the variant cells.

## Results

SSEA3 positive and negative fractions of low passage karyotypically normal (Normal, N^3+^ or N^3-^) and late passage, culture adapted ((47,XX,add(1)(p1)add(1)(q4),der(6)t (6;17)(q27;q1)) (Adapted, A^3+^ and A^3-^) H7 human ES cells (sublines H7.s14 and H7.s6, respectively) were isolated by FACS. As before [4], the clonogenic cells in the Normal subline were predominantly in the N^3+^ subset, being 30 fold less frequent in the N^3-^ subset. In contrast, the cloning efficiency increased for the Adapted cells, and there were substantial numbers of clonogenic cells in both the A^3+^ and A^3-^ subsets (S1 Fig). Groups of 10, 100 and 1000 cells and 20–25 individual cells from each sorted subset—N^3+^, N^3-^, A^3+^ and A^3-^ —were then analyzed by qPCR for their expression of 96 genes, using the TaqMan Stem Cell Pluripotency Assays, (see Experimental Procedures). At the population level, the gene expression patterns of each of these four subsets of cells were comparable to those previously observed for these same subsets of H7 ES cells [4], whether based upon direct analyses of cell populations, or aggregation of single cell data (S1 Fig). In addition, as described in our previous study [4] XIST was downregulated, overall, in the H7.s6 adapted cells (S2 Fig) However, among the individual cells there was substantial heterogeneity in gene expression patterns, both between and within these SSEA3-defined subsets (S1 and S2 Data), indicating that the single cell analysis revealed features that are not evident from population level analyses.

**Fig 1:**
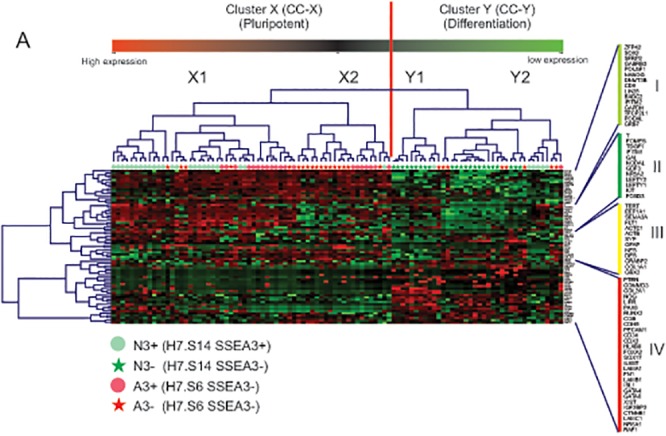
Unsupervised two-way cluster analysis of single cells analyzed by Taqman stem cell pluripotency assays . Samples are plotted horizontally and Taqman probe sets used to interrogate the single cells are displayed vertically. The vertical red line separates the two major clusters identified in the dataset. Green on the heat map indicates high Delta Ct values i.e. low gene expression.

To explore this further, we subjected the single cell data to unsupervised two-way clustering of cells and gene expression (Fig 1). Two broad cell clusters (CC) were apparent, labeled CC-X and CC-Y, each of which could be broken into two subclusters, X1 and X2, Y1 and Y2, respectively, while five distinct gene clusters (GC) were identified, GC-I, GC-II, GC-III, GC-IV and GC-V. GC-V was excluded from the heatmap displayed in Fig 1 and further analysis due to the very low to effectively zero expression of the genes in this cluster. In the Normal cells, CC-X and CC-Y approximated to the subsets defined by the presence or absence of SSEA3 expression, respectively, with 80% of the N^3+^ cells in CC-X and 100% of the N^3-^ cells in CC-Y (Fig 1). Inspection of the gene expression patterns associated with these cell clusters shows that CC-X was mostly linked to relatively high expression of genes in GC-I and GC-II, which include many of the genes characteristically associated with undifferentiated ES cells (e.g. *NANOG*, *POU5F1*, *SOX2*), but low expression of many genes in clusters GC-III, and particularly GC-IV, which include genes associated with various pathways of differentiation. These GC-III and -IV genes tended to be more highly expressed in CC-Y cells, which therefore most likely represent differentiated cells or cell committed to differentiation. There were, however, a small group of N^3+^ cells who fells into the CC-Y cluster and who appeared to have differential expression of GC-II genes such as T, EOMES etc. and may represent N3+ cells closer to commitment than their counnterparts in CC-X. The distinction between CC-X and CC-Y cells is also supported if genes well recognized to be associated with undifferentiated stem cells or various differentiated derivatives are considered (S3 Fig). On the other hand, the Adapted cells behaved differently, with CC-X being associated with cells either expressing or lacking SSEA3: 95% of the A^3+^ cells were in CC-X, while 69% of the A^3-^ subset also comprised CC-X cells (Fig 1). These results are consistent with our previous observation that, although loss of SSEA3 is associated with differentiation in Normal human ES cells, this relationship may be altered upon culture adaptation, with a significant proportion of Adapted cells losing SSEA3 expression but remaining in the undifferentiated stem cell compartment. Further, although the Normal CC-Y subset included many CC-Y1 and CC-Y2 cells, the Adapted CC-Y cells were almost all from the CC-Y2 subcluster, suggesting that culture adaptation influences not only the propensity of ES cells to differentiate, but also their pattern of differentiation.

Considering the two principal CC-X subclusters, CC-X2 was almost exclusively associated with the Adapted cells, which comprised 20 out of 21 CC-X2 cells analyzed (Fig 1). Among these Adapted cells, CC-X2 included both A^3+^ and A^3-^ cells, but was more associated with cells lacking SSEA3–62% A^3-^ versus 33% A^3+^. The one remaining CC-X2 cell was from the N^3+^ fraction. By contrast, CC-X1 was more associated with the Normal cells and cells expressing SSEA3–56% N^3+^, 32% A^3+^ but only 12% A^3-^ (Fig 1). Thus the single cell gene expression data identified novel substates that are related to, but not congruent with those identified by expression of SSEA3 and population level gene expression studies. These novel substates were also altered upon culture adaptation. In particular, CC-X2 tended to be associated with the SSEA3(-) cells that are found within the stem cell compartment and are much more common in cultures of Adapted cells.

CC-X1 and CC-X2 were mainly defined by differential expression of genes in GC-I and GC-II. In particular, GC-I genes tended to be expressed across both sub-clusters, whereas GC-II genes tended to be expressed at higher levels in CC-X1 and lower levels in CC-X2, though there remained substantial heterogeneity between individual cells (Fig 1). It is notable that GC-II includes several genes that are commonly associated with late epiblast and gastrulation in mammals, such as *BRACHYURY*, *EOMES*, *LEFTY1*, *LEFTY2*, *NR5A2* and *NODAL*, suggesting that CC-X2 cells may be less ‘epiblast-like’, or ‘primed’, than CC-X1 cells. A corollary is that culture adaptation has tended to push the population towards a more naïve (CC-X2) state.

**Fig 2:**
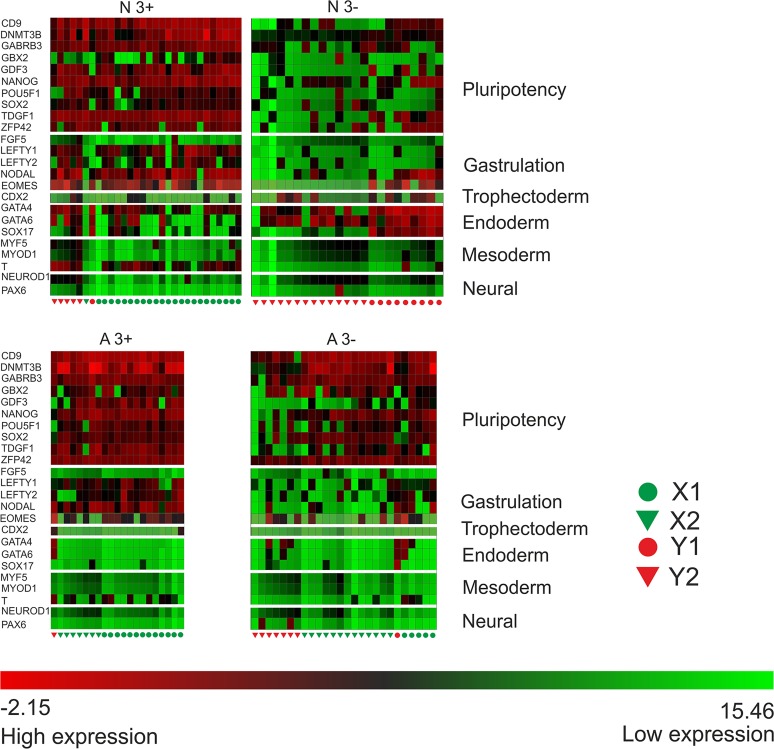
Simultanious gene expression in normal and abnormal SSEA3 + and − fractions. Visualization of simultaneous gene expression in single Normal H7.s14 cells from the SSEA3 positive and negative fractions (N3^+^, N3^-^ respectively) and Adapted H7.s6 cells from the SSEA3 positive and negative fractions (A3^+^ A3^-^ respectively) for selected genes from the Human Stem Cell Pluripotency assays associated with (i) the undifferentiated pluripotent state (*CD9*, *DNMT3B*, *GABRB3*, *GBX2*, *GDF3*, *NANOG*, *POU5F1*, *SOX2*, *TDGF1*, *ZFP42*), (ii) gastrulation (*FGF5*, *LEFTY1*, *LEFTY2*, *NODAL*, *EOMES*), (iii) trophoectoderm (*CDX2*), (iv) Endoderm (*GATA4*, *GATA6*, *SOX17*) (v) Mesoderm (*MYF5*, *MYOD1*, *BRACHYURY*) and (vi) Neural (*NEUROD1*, *PAX6*). The color of each square represents a single genes’ expression (Delta Ct) in a particular cell. Green indicates high Delta Ct values i.e. low gene expression.

Although this unsupervised analysis of the single cell gene expression data identifies obvious clusters of cells that relate to those defined by other criteria (e.g. surface antigen expression, clonogenicity and culture adaptation), implying some degree of co-ordination at the population level, there is marked heterogeneity of gene expression in individual cells suggesting that the co-ordination is far from complete. For example, among the trio of pluripotency-associated transcription factors, *POU5F1OCT4*, *NANOG* and *SOX2*, cells expressing high levels of *POU5F1* but low levels of *NANOG* and/or *SOX2*, or vice versa can be found. The same observation holds when other genes are also considered, whether those typically associated with pluripotent stem cells, or those associated with different stages of embryonic development or different germ layers (Fig 2). Most notably genes typically associated with endoderm, mesoderm and ectoderm lineages were often expressed in the undifferentiated Normal cells—i.e. the CC-X N^3+^—irrespective of their expression of individual genes associated with pluripotency or gastrulation, suggestive of multi-lineage priming. By contrast, this phenomenon was much less evident in the Adapted cells, whether A^3+^ or A^3-^, again suggesting that adaptation tends to push the cells back from a more primed to a more naïve state.

**Fig 3:**
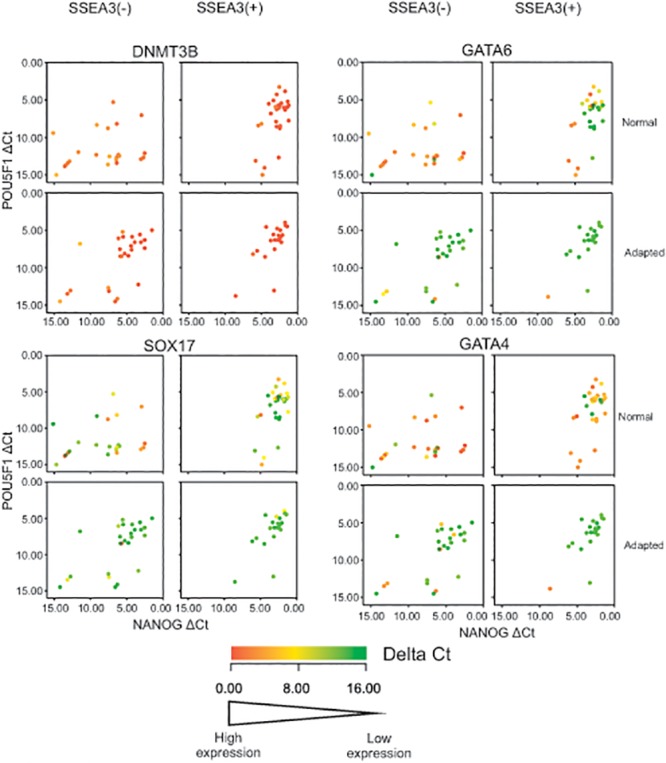
Two-way scatter plots of *POU5F1* (*OCT4*) expression versus *NANOG* expression in the SSEA3 positive and negative assayed fractions from the Normal and Adapted H7 human ES cells. The genes plotted are *DNMT3B*, *GATA4*, *GATA6* and *SOX17*. Each cell is represented by an individual dot and colored according to the Delta Ct value of the genes indicated in the legend appended to each graph. Increasing Delta Ct indicates decreasing expression. Note that, in contrast to the relatively uniform expression of DNMT3B in all cells in each sorted population, the expression of GATA4, GATA6 and SOX17 is quite distinct in most cells of the SSEA3(+) Normal population but in almost no cells in the SSEA(3+) Adapted population.

The contrast between the Normal and Adapted cells with respect to lineage associated genes was most striking in the case of genes associated with endoderm differentiation, notably *GATA4*, *GATA6* and *SOX17*. When the single cell data is represented in a scatter plot of expression of the two characteristic stem cell marker genes, *POU5F1* and *NANOG*, a clear cluster of cells expressing these genes is evident in the N^3+^ and A^3+^ populations, and persists in the A^3-^ populations, but is lost in the N^3-^ population (Fig 3). If it is posited that these *POU5F1/NANOG* expressing clusters represent the undifferentiated stem cells, the observations are consistent with the conclusions above and in our earlier study [4] that many A^3-^ cells remain within the stem cell compartment whereas the N^3-^ cells have initiated differentiation. However, when the expression of other genes are examined, it is clear that many of the *POU5F1/NANOG* expressing N^3+^ cells also express one or more of the endoderm-related genes, *GATA4*, *GATA6* and *SOX17*. Again there was no evident co-ordinate expression of these genes. On the other hand they are almost not expressed in the A^3+^ cells or the A^3-^ cells, although many cells in the N^3-^ population express them. As we have previously suggested, one of the selective advantages that could drive adaptation is the loss of an ability to generate endoderm, which is a potential source of factors such as BMPs that may drive further differentiation of persisting undifferentiated stem cells [9]. The present results suggest that the loss of a propensity for endoderm reflects changes to the dynamics of substates within the stem cell compartment and the elimination of an endoderm primed substate.

## Discussion

The results of our present single cell transcriptome study are consistent with our earlier population based study of early passage, Normal, and late passage, Adapted, human ES cells, and confirm that the surface antigen SSEA3 defines substates, the dynamics of which are altered during culture adaptation [4]. Unlike the expression of many markers that have been used to identify substates of pluripotent stem cells, the SSEA3 epitope is not the product of a single gene, as it comprises a set of carbohydrate moieties of the glycosphinogolipid, sialylα2-3galβ1-3globoside [23–25]. Further, whether cells are scored as SSEA3(+) or (-) appears to depend upon how that glycolipid is displayed rather than only its presence or absence [25]. Consequently, although we do not have a clear understanding of the molecular mechanism of SSEA3 positivity, we suggest that it provides a signal that integrates a wide array of transcriptomic and metabolomic information about cell state. Certainly various studies, including the current results, indicate that the scoring of cells as SSEA3(+) and (-) does have functional correlates [26] [25] [27] [4] [28].

Broadly, the single cell clustering analysis indicated that all of the cells can be grouped into two clusters corresponding to undifferentiated stem cells, CC-X, which express genes classically associated with pluripotent stem cells, such as *ZFP42*, *LIN28*, *CD9*, *POU5F1*, *SOX2* and *NANOG*, and differentiated cells, CC-Y, which do not express significant levels of these genes but did express various genes associated with differentiation (S3 Fig). Whether from the cluster analysis (Fig 1), or by consideration of two key markers of undifferentiated pluripotent stem cells, *POU5F1* and *NANOG*, (Fig 3), the transcriptomes of the N^3-^ cells, all of which belong to Cell Cluster CC-Y, are consistent with them having left, or being in the process of leaving the stem cell compartment, as we argued before based on population level transcriptome data and on functional, clonogenicity assays [4]. By contrast, all the other subsets of Normal and Adapted cells contained undifferentiated, CC-X cells, though in a decreasing proportion from A^3+^ to N^3+^ to A^3-^. However, these data also exposed marked heterogeneity within the cell clusters from which two observations stand out.

First, a subset of the cell cluster CC-X, CC-X2, was almost exclusively associated with the culture adapted cells. It has been widely proposed that human ES cells correspond to an epiblast like state, resembling murine EpiStem cells, which has also been called a primed state [29, 30] [31, 32], although several papers have reported culture conditions whereby human ES cells adopt some characteristics of more naïve, murine ES cells [33–37]. However, the heterogeneity between individual human ES cells in our current study suggests that populations of these cells may exist as distributions along a naïve-primed axis. Moreover, since the CC-X2 cells are primarily defined by a tendency to low expression of genes typically associated with the epiblast embryonic state, such as *BRACHYURY*, *EOMES*, *LEFTY1*, *LEFTY2*, *FOXD3*, this result is consistent with the notion that culture adaptation leads to the cells adopting a more naive-like state. In addition, the adapted cells as in our previous study [4] had very low expression of XIST. Lack of X-inactivation has been described as a feature of naïve pluripotent stem cells (reviewed in [38]) and therefore would be anticipated in any naïve-like cells present in human PSC cultures. However, gains of an X chromosome are commonly reported in hES cell lines (see review in [1]; [7]) and may represent an independent change brought about by the cells being cultured under strongly selective conditions. Recently, the phenomenon of ‘erosion’ of X-inactivation in human pluripotent stem cells has been reported [39], again suggesting that changes in the inactivation of the X-chromosome may be due to selection rather than a shift to a naive-like state.

Counterintuitively, despite the simple view that associates loss of SSEA3 with priming to differentiate [4], the CC-X2 cells tended to be SSEA3(-), although not exclusively so. On the other hand it is notable that mouse ICM cells, which correspond to the naïve condition of murine ES cells are also SSEA3(-). The observation that hPSCs exist in a range of states within the stem cells compartment, with some cells in more ‘naïve-like’ state concurs with recent single-cell data that utilized a different set of cell surface markers as discriminants and concluded that heterogeneity may be an inherent feature of hPSCs in culture[40].

Second, several of the N^3+^ cells expressed genes such as *GATA4*, *GATA6* and *SOX17*, suggesting that these cells are primed for endoderm differentiation, whereas cells expressing these genes were notably absent from the A^3+^ subset. Mutations in variant stem cells could provide growth advantages not only by affecting cell division or cell survival but also by altering their pattern of differentiation since derivative cells may form a niche that can influence the behavior of persisting stem cells. For example, Bendall et al [41] have reported that fibroblasts derived from human ES cells drive the further proliferation of the remaining human ES cells by producing IGF2 in response to exogenous FGF. We have previously reported that culture adapted H7 human ES cells have a much reduced propensity for endoderm differentiation [9]. This may be a source of selective growth advantage since endoderm is known to produce BMP, a factor that induces human ES cell differentiation so that spontaneous endoderm differentiation can lead to a positive feedback loop that rapidly depletes the pool of undifferentiated cells [42]. Our present results suggest that the reduced tendency for endoderm differentiation of the culture adapted human ES cells arises from altered dynamics of substates of cells within the stem cell compartment prior to commitment to differentiation. Likewise we previously concluded in another system, neuronal differentiation of NTERA2 EC cells induced to differentiate with retinoic acid, that the eventual fate of the differentiated cells depended upon the behavior of lineage biased substates within the stem cell compartment prior to induction of differentiation [17].

This study highlights the increasing importance of analyzing cells at a single cell level rather than relying of average measures from population analyses. The results are consistent with the view that within the stem cell compartment human ES cells may transiently occupy different substates. Further, since cells in these substates express different patterns of gene expression, it seems likely that, when subjected to differentiation signals, they may exit the pluripotent state on different differentiation trajectories thus leading to heterogeneous differentiation [15]. Although the heterogeneity in expression of genes such as GATA4, GATA6 and SOX17 could be due in part to differential expression through the cell cycle [43], the lack of coordinate gene expression between lineage markers in single cells (Fig 2) suggests that the substates we have inferred reflect other attributes of cellular regulation. Understanding the mechanisms that control the dynamics of these stem cell substates therefore has implications for seeking culture conditions to minimize the appearance of genetically variant cells, by reducing the selective advantages associated with some biased differentiation potentials. Likewise it has implications for approaches to controlling the differentiation of ES cells to specific cell types for particular applications.

## Materials and Methods

### Human ES cell culture

Two sublines of the human ES cell line, H7 [44], were used in the current study [3] [4]. One subline, designated H7.s14, has a normal female karyotype (46, XX) and was used at a passage level less than 40. The second subline, designated H7.s6, has an abnormal karyotype (47,XX,add(1)(p1)add(1)(q4),der(6)t (6;17)(q27;q1)), and was used at a passage level in excess of p130. The cells were cultured on mitomycin C inactivated mouse embryonic fibroblasts derived from e12.5 MF-1 mouse embryos, in Knockout DMEM (Invitrogen), supplemented with 20% Knockout Serum Replacement (Invitrogen) and 4 ng/ml bFGF (Peprotech), as previously described[3]. The abnormal line, H7.s6, shows characteristics of culture adaptation, such as increased population growth rate and cloning efficiency, compared to the normal, H7.s14 line.

### Cell sorting and single cell isolation

Cell were harvested with trypsin:EDTA, stained by indirect immunofluorescence for the expression of the ES cell surface antigen, SSEA3, using pre-titered supernatant from hybridoma MC-631 [26] and FITC-conjugated goat anti-mouse IgG+IgM (Caltag), as previously described [4, 27]. Following staining, the cells were analyzed by flow cytofluorimetry, and sorted using a Dakocytomation Flow Cytometer. Specified numbers of cells from antigen-positive and antigen-negative subpopulations were deposited into the wells of a 96-well thermal cycling plate, in 0.5μl of sorting buffer. Pre-sort and post-sort buffer samples were collected into the plates as negative controls.

### Cell Lysis and reverse transcription

Following sorting, the human ES cells were lysed using the TaqMan gene expression Cells-to-CT^TM^ kit (Ambion Cat No. AM1728). Lysis solution (4.1μl) with DNase was added to single cells in 96-well plates and incubated for 5 min at room temperature. The reaction was terminated with 0.5μL of stop solution. Cell lysates were frozen at -80°C. Then reverse transcription reagents (RT) were added to the 96-well plate containing the single cell lysates. The RT reaction contained 40% lysate for the single cell samples and was performed at 37°C for 60 min followed by a 95°C heat inactivation step for 5 min. (See S6 Fig for a summary of the lysis, reverse transcription, pre-amplification and qPCR protocol)

### Multiplex preamplification

The resulting cDNA in a volume of 12.5μl was used for multiplex preamplification with a TaqMan PreAmp Pool for the Human Stem Cell Pluripotency assay panel, with 95 specific cDNA targets [45], Life Technologies Cat. No. 4414077). The preamplification was performed by incubating at 95°C for 10 min followed by 14 cycles at [95°C for 15 sec, 60°C for 4 min] in a Applied Biosystems GeneAmp PCR System 9700.

### QC of pre-amplified product

A preliminary Real-Time PCR experiment was performed to distinguish wells containing single cells from empty wells. The preamplified product was diluted (1:128) and mixed with TaqMan Gene Expression Master Mix (Life Technologies PN 4370074) to measure expression of 3 targets (Life Technologies *NANOG* assay ID Hs02387400_g1, *ACTB* assay ID Hs99999903_m1, and *GAPDH* assay ID Hs99999905_m1). The Ct measurement was used to confirm that the well contained a cell. Samples with Ct values of *ACTB* and *GAPDH* that showed positive expression and good correlation were further tested.

### Quantitative Real-Time PCR

Preamplified product was diluted (1:128), and quantitative real-time PCR of 95 targets in single-plex using duplicates was performed. The targets used were described previously [45]. qPCR was performed with Human TaqMan Stem Cell Pluripotency assays (Life Technologies Cat. No. 4385344) in 384-well plates amplified in Applied Biosystems 7900HT Fast Real-Time PCR System. Raw cycle threshold values from each sample preparation for *GAPDH* and beta-ACTIN *(ACTB)* were used to identify data points lying greater than one standard deviation from the fraction population mean expression value. In sample preparations from single cells, those with higher or lower Ct values than one standard deviation of the population mean generally had correspondingly higher or lower Ct values for two other control genes *CTNNB1* and *RAF1*. These samples were excluded from further analysis as likely to contain damaged/ dying cells or multiple cells respectively. All subsequent gene expression data were expressed as the difference between Ct values for individual genes and the corresponding Ct value for *ACTB* obtained on the same cells (DeltaCt_ACTB_).

Hierarchical clustering by genes and cells and heat mapping was created using Genesis [46] by Pearson correlation distance with complete linkage.

To test the feasibility of examining the gene expression of individual cells, single cells, and multiples of 10, 100, 1000 and 10,000 cells from the N3(+), N3(-), A3(+) and A3(-) subsets were deposited directly following FACS into the wells of a 96-well plate and then examined by TaqMan qPCR for *ACTB*, *GAPDH* and *NANOG*. The expression of *ACTB*, *GAPDH* and *NANOG* in preparations of 10,000, 1,000, 100, 10 and single cells from each subset showed a stepwise decrease (i.e. increase in Ct value), with Ct values that were inversely proportional to the log cell number in each preparation (S4 Fig), indicating that the assay was sufficiently sensitive to read out changes in expression from single cells. In the case of the single cell preparations, samples were excluded from further analysis if their ACTB/GAPDH expression values fell outside one standard deviation of the mean (S5 Fig). Most of these outlier samples also expressed correspondingly high or low levels of the additional two control genes, β-catenin (*CTNNB1*) and *RAF1*, suggesting that they either contained multiple cells or cells undergoing apoptosis or necrotic death.

The overall gene expression patterns observed in the samples prepared from the different subsets of H7 cells were comparable to those found in our earlier transcriptome analysis of SSEA3-sorted subsets of the same sublines of H7 ES cells, analyzed by Affymetrix microarray [4]. When compared to our previous study, similar relative levels of gene expression were noted in the present study, e.g. for the pluripotent signature genes *NANOG*, *POU5F1*, *TDGF1*, *GABRB3*, *DNMT3B* and *GDF3* (S1 Fig). Taken together, these observations suggest that the expression data derived from the TLDA analysis provide a reliable picture of the transcriptome of the different subsets of H7 cells isolated in these experiments, broadly consistent with the results of previous population level based studies.

## Supporting Information

S1 Data Delta Ct values, normalized to beta-actin (ACTB). (XLS)

S2 Data Raw Ct values. (XLS)

S1 Fig Bulk comparison of Normal and Adapted SSEA3 sorted fractions. **Panel A:** Single cell re-plating efficiencies of H7.s14 and H7,s6 SSEA3 positive and negative cells. **Panel B,** Comparison of selected pluripotency associated genes from Affymetrix gene microarray derived expression values from Enver et. al. [4] and the Delta Ct (normalized to beta-actin (ACTB)) expression values from the analysis of 1000 sorted cells run on TaqMan assays in this study. All values are expressed as normalized values to the H7.s14 SSEA3+ sample.
(EPS)

S2 Fig Two-way scatter plots of *POU5F1* (*OCT4*) expression versus *NANOG* expression against *XIST* in the SSEA3 positive and negative two assayed fractions from the Normal and Adapted H7 human ES cells. Each cell is represented by an individual dot and colored according to the Delta Ct value of *XIST*. Increasing Delta Ct indicates decreasing expression.
(EPS)

S3 Fig Average gene expression between clusters X and Y. The average gene expression (Delta Ct) of genes known to be generally associated with pluripotency of human ES cells (*CD9*, *DNMT3B*, *FGF4*, *GABRB3*, *GAL*, *GBX2*, *GRB7*, *LEFTY1*, *LEFTY2*, *LIFR*, *LIN28*, *NANOG*, *NODAL*, *NOGGIN*, *PODXlL*, *POU5F1*, *SOX2*, *TDGF1*, *TERT*, *ZFP42*) compared to differentiation associated genes (*CDX2*, *DDX4*, *EOMES*, *FGF5*, *FOXA2*, *GATA4*, *GATA6*, *HLXB9*, *NEUROD1*, *PAX4*, *PAX6*, *SOX17*, *T*) between the two major clusters (X and Y) identified in Fig 1.
(EPS)

S4 Fig Evaluation of single cell TaqMan real-time PCR for sensitivity and outliers. Gene expression values for ACTB (beta-actin), GAPDH on single cells, 100, 1000 and 10000 cells sorted for H7.s14 SSEA3+, H7.s14 SSEA 3-, H7.s6 SSEA3+, H7.s6 SSEA3 -. ‘NTC’ indicates RNA extracted from a given fraction but not transcribed to cDNA.
(EPS)

S5 Fig Two-way plots of GAPDH expression versus ACTB expression in the four assayed fractions compared to the control genes *CTNNB1* and *RAF1* from the human pluripotency low density array [45]. Each cell is represented by an individual dot and colored according to the Delta Ct value of the genes indicated in the legend appended to each graph. Increasing Delta Ct indicates decreasing expression. The shape of each point indicates whether a data point falls within one standard deviation of the population mean for each fraction.
(EPS)

S6 Fig Summary of the protocol used to analyze sorted single cells using TaqMan assays. (EPS)

S1 Table List of genes contained in each cluster identified in Fig 1A. Genes colored green indicate an average expression value of < 10 Delta Ct(ACTB) in undifferentiated samples in the ISCI project dataset [45].
(DOCX)
