# Supplementary material for: Culture Adaptation Alters Transcriptional Hierarchies among Single Human Embryonic Stem Cells Reflecting Altered Patterns of Differentiation
Source: PLoS One. 2015 Apr 14;10(4):e0123467. doi: 10.1371/journal.pone.0123467 (PMC4397016; doi:10.1371/journal.pone.0123467)
Supplement: S1 Table — Genes colored green indicate an average expression value of < 10 Delta Ct(ACTB) in undifferentiated samples in the ISCI project dataset [45]. (DOCX) [file pone.0123467.s009.docx]

| **Cluster** | **Genes** |
| --- | --- |
| I | **ZFP42**, SOX2, **SFRP2**, **GABRB3**, **POU5F1**, **NANOG**, **DNMT3B**, **CD9**, **LIN28**, **BXDC2**, **IFITM2**, **GAPDH**, TFCP2L1, **PODXL**, **GRB7** |
| II | T, **EOMES**, **TDGF1**, **IFITM1**, **GAL**, **NODAL**, **GDF3**, **NR5A2**, **LEFTY2**, **LEFTY1**, **KIT**, **FOXD3** |
| III | **TERT**, **EEF1A1**, **FLT1**, **ACTC**, **ACTB**, **SYP**, GFAP, **NES**, DES, **CRABP2**, **COL1A1**, GBX2 |
| IV | **PTEN**, COMMD3, **COL2A1**, **NOG**, LIFR, PAX6,RUNX2,CGB,CDH5,PECAM1,CD34,CDX2,HLXB9,**FOXA2**,**SOX17**,**IL6ST**,**LAMA1**,**FN1**,**LAMB1**,ISL1,**GATA4**,**GATA6**,XIST,**IGF2BP2**,**CTNNB1**,**LAMC1**,**NR5A1**,**RAF1** |
